# Monoclonal Antibodies as a Breakthrough in Personalised Leukaemia Therapy: What Pharmacists and Doctors Should Know

**DOI:** 10.3390/pharmacy13060169

**Published:** 2025-11-20

**Authors:** Anastasiia Ryzhuk, Sergiy M. Kovalenko, Marine Georgiyants, Kateryna Vysotska, Victoriya Georgiyants

**Affiliations:** 1 Pharmaceutical Chemistry Department, National University of Pharmacy, 61002 Kharkiv, Ukraine; ryzhuk.anastasia@gmail.com; 2 Department of Organic Chemistry and Molecular Materials, V. N. Karazin Kharkiv National University, 61022 Kharkiv, Ukraine; 3 Department of Anesthesiology, Intensive Care and Pediatric Anesthesiology, Kharkiv National Medical University, 61022 Kharkiv, Ukraine; mgeorgiyants@gmail.com; 4 Department of Propaedeutics of Internal Medicine and Physical Rehabilitation, V. N. Karazin Kharkiv National University, 61022 Kharkiv, Ukraine; ekaterinalipakova@gmail.com

**Keywords:** monoclonal antibodies, side effects, pharmaceutical aspects, leukaemia, oncology pharmacy, hospital pharmacy, blinatumomab, inotuzumab ozogamicin, gemtuzumab ozigomicin, rituxan, rituximab, ofatumumab, obiuntuzumab, alemtuzumab, acute lymphocytic leukaemia, acute myeloid leukaemia, chronic lymphocytic leukaemia, chronic myeloid leukaemia

## Abstract

Monoclonal antibodies (mAbs) are an important medical innovation in modern medicine. They are an effective therapy for several subtypes of leukaemia but may have undesirable effects, which may be minimised through the provision of interdisciplinary care including a pharmacist. The goals of this narrative review were twofold: first, to summarise the literature on the side effects of mAbs and the challenges of their preparation, and to provide recommendations for the safe preparation of mAb drug formulations for clinicians. Second, to suggest clinical roles for pharmacists to improve patient safety and clinical outcomes for leukaemia patients receiving mAb therapy. The review covers data from 178 scientific and official sources of information on the types of targeted immunobiological drugs for the treatment of various types of leukaemia. The results are a detailed description of the possible side effects from mAb therapy and a list of suggested actions that can be taken to prevent them. Pharmaceutical aspects of the use of mAbs, such as pharmacoeconomics, compounding and stability, are also discussed. The discussion is organised according to the current classification of leukaemia. The drugs considered include blinatumomab, inotuzumab ozogamicin, gemtuzumab ozogamicin, rituximab, ofatumumab, obinutuzumab, and alemtuzumab. The review offers a comprehensive resource to equip pharmacists and other clinicians to optimise mAb therapy and promote the safe use of these novel therapies.

## 1. Introduction

Monoclonal antibodies (mAbs) are an important medical innovation in modern medicine. They have proven efficacy in the treatment of cancer [1,2,3,4]. mAbs are increasingly being investigated in clinical trials [5]. The number of registered interventional studies using these drugs in the treatment of malignant diseases was high at the end of the twentieth century, thanks to the approval of the first mAbs-based cancer treatment.

Their global market places a significant burden on healthcare systems and hampers access to medicines, with low- and middle-income countries experiencing limited or no availability [6,7,8].

**Figure 1:**
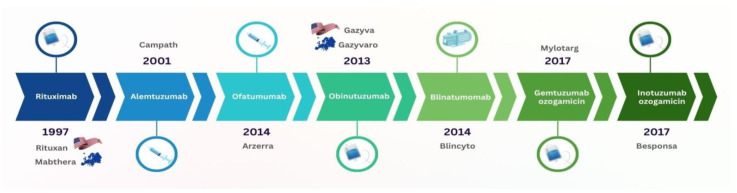
Chronology of mAbs introduction in the treatment of leukaemia.

Leukaemia has always been one of the most pressing onco-haematological pathologies, with a pronounced global spread, rapid progression, and high risk of mortality [9,10]. Rituximab [11] is the first mAb registered by the U.S. Food and Drug Administration (FDA) for the treatment of B-cell chronic lymphocytic leukaemia (B-CLL), which was a breakthrough not only for leukaemia therapy but was also the world’s first registered targeted immunobiological cancer drug. The successful introduction of rituximab therapy contributed to the further discovery of new drugs for other forms of leukaemia [12] (Figure 1).

**Figure 2:**
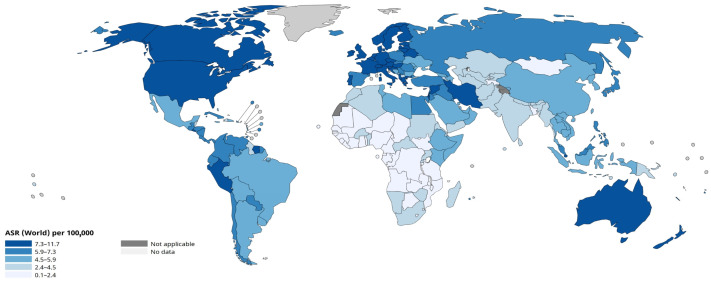
Age-standardised incidence of leukaemia in the world (per 100,000 population). Adapted from WHO, 2022. URL: https://gco.iarc.who.int/today/en/dataviz/maps-heatmap?mode=population&cancers=36 (accessed on 14 October 2025) [13].

However, the progress in the development and implementation of targeted therapies (TT) has not yet been able to stop the progression of the disease. According to the World Health Organisation (WHO), in 2022, a significant number of people were diagnosed with leukaemia worldwide, covering all geographical regions. Incidence rates ranged from less than 2.4 to more than 7.3 cases per 100,000 people, depending on the country (Figure 2), but no country was left untouched by this disease. This indicates the widespread nature of the disease, which is recorded both in countries with highly developed medical systems and in regions with limited access to medical care.

**Figure 3:**
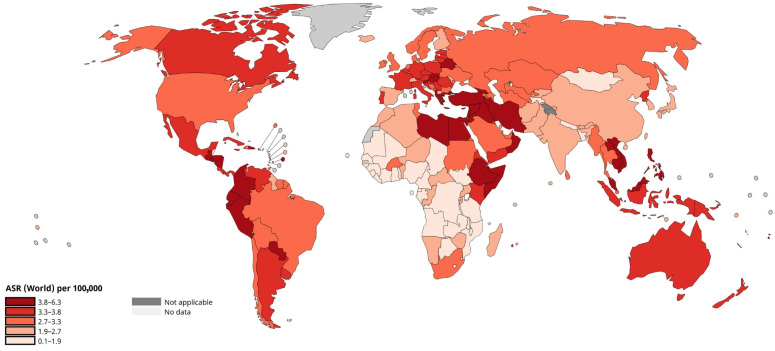
Age-standardised leukaemia mortality rate in the world (per 100,000 population). Adapted from WHO, 2022. URL: https://gco.iarc.who.int/today/en/dataviz/maps-heatmap?mode=population&cancers=36&types=1 (accessed on 14 October 2025) [15].

A similar situation is observed with mortality rates (Figure 3). Despite the availability of modern diagnostic and treatment methods, leukaemia remains the cause of a significant number of deaths, even in countries with a high level of healthcare development. In countries with poor medical infrastructure, the mortality rate from this disease is significantly higher, due to late diagnosis and limited access to specialised therapies, including stem cell transplantation and TT [9,10,14].

In addition to high morbidity and mortality rates, another problem with leukaemia is therapy. As mentioned above, mAbs-based drugs are currently the newest class of treatment for this disease. Pharmacists specialising in oncology are an integral part of multidisciplinary teams, helping to optimise therapy and improve patient compliance with treatment regimens. Their interventions improve the clinical and economic outcomes of cancer treatment. Data from systematic reviews show that the involvement of pharmacists in the management of cancer patients increases treatment adherence and reduces the incidence of side effects and medication problems [16,17,18,19]. The lack of interaction between a doctor and a pharmacist during therapy may result in improper dosage selection, failure to account for individual patient characteristics, increased risk of complications, and worsened quality of treatment. In addition, the preparation of mAbs is a technologically complex process that requires strict adherence to dilution, sterility, and correct dosage. Errors or negligence at this stage can lead to a decrease in the efficacy of the drug or increased toxic reactions [19,20,21,22].

According to the FDA, the most common side effects of mAb-based drugs in the treatment of various types of leukaemia included: infusion-related reactions, cytokine syndrome, neurotoxicity, thrombocytopenia, and infectious complications [23,24,25,26,27,28,29].

Thus, although mAbs therapy opens up new prospects, it does not provide ideal results in terms of either reducing the incidence or improving survival.

In this review, we asked the question following: how can the existing shortcomings of mAb-based drugs be eliminated and the treatment of various types of leukaemia improved? The aim was to comprehensively review the available data on the use of mAbs in leukaemia therapy, with a focus on aspects within the area of responsibility for pharmacists who prepare and dispense these medications as well as clinical pharmacists who collaborate with physicians to prescribe or monitor therapy. A brief description of targeted immunobiological therapy is provided in terms of their mechanisms of action and effectiveness for various forms of leukaemia to aid understanding of the approaches to effective pharmaceutical care and administration of mAb drugs for the treatment of various types of leukaemia.

Particular attention is paid to the safety profile of the drugs and the practical aspects of their use—preparation, storage conditions, stability, and the need for premedication. The pharmacist’s role in an interdisciplinary approach to treatment is also emphasised.

## 2. Materials and Methods

This narrative literature review aimed to summarise knowledge and data about the mechanism of action, side effects, administration, and pharmacoeconomic aspects of mAbs-related pharmaceuticals for leukaemia patients. A targeted search was carried out across PubMed, Web of Science, Sciencedirect, Google Scholar, and local scientific journals and covered a wide range. The search did not exclude early publications, but the focus was on recent years. Various combinations of the following keywords were used in the search: “pharmacist”, “clinical pharmacist”, “compounding”, “oncology pharmacy”, “therapy”, “monoclonal antibodies”, “side effects”, “monoclonal antibodies”, “leukaemia”, “blinatumomab”, “inotuzumab ozogamicin”, “gemtuzumab ozigomicin”, “rituxan”, “rituximab”, “ofatumumab”, “obiuntuzumab”, “alemtuzumab”, “acute lymphocytic leukaemia”, “acute myeloid leukaemia”, “chronic lymphocytic leukaemia”, and “chronic myeloid leukaemia”.

There were no exclusions based on article type, and conference abstracts were also included. FDA and EMEA guidance documents were used to summarise practical information. The authors read the titles and abstracts to select those relevant to clinicians (M.G.) and pharmacists (V.G.). The selected articles were read in full, and relevant information was extracted. In addition, the bibliographies of the included articles were analysed to search for sources. Preference was given to the literature from the last 5–10 years, but earlier publications were not excluded if more recent publications were not available.

The review included articles that described in detail the mechanism of action of the drugs, the specifics of its use, and the side effects that arise during therapy. Aspects of pharmaceutical care and clinical data results were highlighted.

To make the results more visual, all figures were created using the Canva Pro (web application, Canva Pty Ltd., Sydney, Australia).

## 3. Results and Discussion

### 3.1. Introduction to Leukaemia Therapy

Leukaemia is a heterogeneous group of malignant haematological diseases characterised by the uncontrolled growth of abnormal white blood cells in the bone marrow, which ultimately leads to their accumulation in the blood and other tissues [30].

Leukaemia occurs as a result of the malignant transformation of pluripotent haematopoietic stem cells. In rare cases, it may also involve more committed stem cells with limited self-renewal capacity [31].

**Figure 4:**
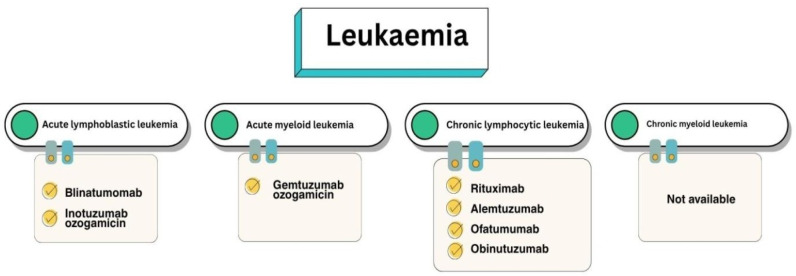
MAbs approved for the treatment of various leukaemia subtypes.

This type of disease can be classified into four main types (Figure 4) depending on the rate of progression of a particular type of white blood cell involved [30,32,33] and therefore the target for a particular mAb.

In acute leukaemia, the peripheral blood or bone marrow is characterised by the presence of more than 20% blasts [31].

Acute lymphocytic leukaemia (ALL) is observed in patients with blast transformation of B and T cells. It occurs due to the excessive production of immature leukocytes. It is the most common leukaemia in paediatric practice. It typically affects children aged 2–10 years [34]. In ALL, a chromosomal translocation or abnormal chromosome number can lead to mutations in lymphoid progenitor cells, which results in lymphoblasts [31]. A bispecific antibody (BsAb), blinatumomab, and a conjugated drug, inotuzumab ozogamicin (InO), are currently approved for the treatment of ALL [35,36,37].

Acute myeloid leukaemia (AML) is characterised by more than 20% myeloid blasts and is the most common acute leukaemia in adults. It is an aggressive cancer with a variable prognosis depending on the molecular subtypes [31]. It differs from ALL in the presence of Auer bodies [34]. Currently, a conjugated mAbs, gemtuzumab ozo-gamicin (GO), is used for AML [38,39,40,41].

Unlike acute leukaemia, chronic leukaemia cells partially mature [42,43]. These partially mature cells do not function effectively and divide too quickly. They accumulate in the peripheral blood and lymphoid organs, which can lead to anaemia and thrombocytopenia, as well as leukaemia [31,42,43].

Chronic lymphocytic leukaemia (CLL) is caused by the proliferation of monoclonal lymphoid cells. CLL is mostly considered a dormant disease, so not all patients diagnosed with it need to start treatment before the onset of symptoms. The largest number of targeted immunobiological drugs have been approved for this type of leukaemia [44,45,46]. Among them are three anti-CD20 drugs: rituximab, ofatumumab, and obinutuzumab. There is also one humanised anti-CD52 drug: alemtuzumab.

Chronic myeloid leukaemia (CML) typically results from the reciprocal translocation and fusion of BCR on chromosome 22 and ABL1 on chromosome 9, which leads to a dysregulated tyrosine kinase on chromosome 22, called the Philadelphia (Ph) chromo-some. This, in turn, causes a monoclonal population of dysfunctional granulocytes, predominantly neutrophils, basophils and eosinophils [31,47,48]. In CML, the use of mAbs is currently limited and there are no officially approved drugs.

TT drugs are the first line of treatment for some types of leukaemia. These drugs find and target specific proteins or changes in cells that cause leukaemia. Unlike chemotherapy (which affects both healthy and diseased cells), TT focuses only on the cellular changes that cause cancer.

### 3.2. Targets of Immunobiological Therapy for B-ALL

ALL is the most common paediatric malignancy [49]. It is believed that ALL occurs after DNA damage leads to the uncontrolled growth of lymphoid cells and their spread throughout the body. Splenomegaly and hepatomegaly occur due to the sequestration of platelets and lymphocytes in the spleen and liver. As the white blood cells are not typical, the spleen reacts to them by trying to remove them from the blood [50].

**Figure 5:**
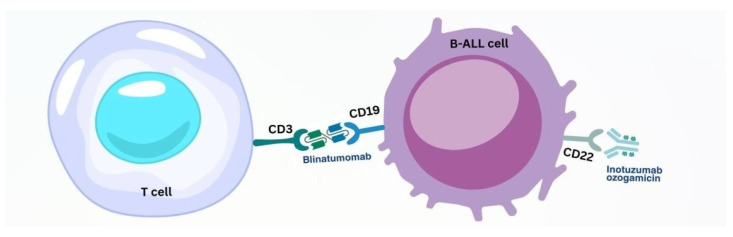
Mechanism of action of mAbs drugs on B-ALL cells.

Treatment of adults with ALL has made tremendous progress over the past 15 years. The successes have been particularly noticeable with B-lineage ALL (B-ALL). The development of BsAbs directed against CD19 has opened a new era in overcoming persistent minimal disease in patients with newly diagnosed ALL, as well as in the successful treatment of patients with relapsed disease. Immuno-conjugates targeting CD22 (Figure 5) have also played an equally impressive role in improving outcomes in these patients. These advances are now being extended to advanced regimens for B-ALL [51].

#### 3.2.1. BsAb Therapy of B-ALL

BsAbs that engage T cells: BsAbs are antibodies that have two different antigen-binding sites, which allows them to bind two antigens simultaneously. The main mechanism of action is the recruitment and activation of T cells to destroy tumour cells. BsAbs promote enhanced activation of T cells, which produce perforin and granzymes. Perforin creates pores in the tumour cell membrane, through which granzymes enter and trigger apoptosis. Blinatumomab, by simultaneously binding CD19 (a characteristic surface marker of B cells) on leukaemic B cells and CD3 (the main marker of T cells involved in signal transduction from the T-cell receptor) on T cells (Figure 5), can mediate a direct cross-talk between T cells and tumour cells, leading to the targeted and highly effective destruction of tumour cells [52].

Blinatumomab is the first antibody approved by the FDA for the treatment of patients with minimal residual disease (MRD) B-ALL [52,53,54]. MRD-positive ALL is a condition where a patient still has leukaemia cells after treatment (e.g., chemotherapy), although they have achieved complete morphological remission. Even a small number of residual cells may indicate a high probability of relapse, so patients with MRD+ may be candidates for new treatments [55,56]. In the BLAST study, blinatumomab demonstrated a 78% complete MRD response after the first cycle [52].

Blinatumomab is also effectively used to treat relapsed or refractory (R/R) B-ALL [57,58,59,60,61]. This form of the disease is characterised by either a return of leukaemia after achieving remission (relapse) or a lack of response to standard therapy from the outset (refractoriness). Patients with R/R B-ALL usually have a worse prognosis and require alternative immunotherapeutic approaches. In a multicentre Phase II study, the drug demonstrated a complete remission rate of 43% after two cycles, with 82% of patients who achieved CR also becoming MRD-negative [62].

Ph-positive ALL is a subtype of ALL characterised by the presence of the Ph chromosome, a genetic abnormality resulting from a translocation between chromosomes 9 and 22 and leading to the formation of the oncogenic protein BCR-ABL. This subtype of leukaemia has unique biological and clinical features. Particular attention is drawn to people aged 15 to 39 years, a patient population that demonstrates significantly worse treatment outcomes and lower survival rates compared to younger children. The treatment of Ph-positive ALL has undergone significant changes in recent years. One of the newest promising methods is treatment with blinatumomab [63]. In patients who had already received tyrosine kinase inhibitors, blinatumomab showed a 36% remission rate and 88% MRD-negativity. In combination with dasatinib (induction without chemotherapy), 98% remission and 81% deep molecular response were achieved [52]. Thus, bilinatumomab has shown promise as an alternative to chemotherapy in the first-line treatment of ALL.

Side effects: Despite its efficacy, the drug has a number of side effects that should be carefully monitored during therapy. Cytokine release syndrome (CRS) and neurotoxicity are the most significant among them. Although it is noted [64] that these side effects are lower than during traditional chemotherapy.

One of the main groups of complications is neurological side effects, which range from mild symptoms such as headache, tremor, confusion, and disorientation to more severe manifestations such as aphasia, seizures, and stupor [65,66]. They usually occur in the first cycle of treatment, with milder forms appearing more rapidly and severe forms having a later onset and shorter duration. The pathogenesis of neurotoxicity is associated with the migration of peripheral T cells to the vascular endothelium and perivascular space, the activation of T cells, and the release of cytokines that damage nervous tissue [64]. For the risk of neurologic events, blinatumomab showed a higher risk of encephalopathy compared to chemotherapy, while no difference was observed for the risk of seizure [64]. To minimise these manifestations, symptomatic treatment is used, including intravenous fluids and anti-inflammatory drugs, and dexamethasone is prescribed at a dose of 8 mg every 8 h [65,66]. In grade 3, blinatumomab is temporarily discontinued until mild symptoms improve, after which treatment can be resumed with a gradual increase in dose. In case of severe grade 4 neurotoxicity or in case of prolonged and recurrent symptoms, therapy is discontinued permanently. Primary seizure prevention is not currently recommended due to the low frequency of seizures, but anticonvulsants, for example, levetiracetam, can be used if necessary [19,67].

Another serious complication of blinatumomab therapy is *CRS,* which occurs as a result of a massive inflammatory response with excessive release of cytokines IL-6, IL-10, and interferon-γ after the activation of cytotoxic T cells [19,68,69,70]. Clinically, this is manifested by fever, chills, haemodynamic instability, and symptoms of capillary leakage. This syndrome occurs in approximately 16% of patients, of whom 5% have a severe form. To prevent severe manifestations, premedication with dexamethasone is performed, and in patients with a high tumour burden, additional cytoreduction with dexamethasone or cyclophosphamide is performed. In the event of severe manifestations, blinatumomab is temporarily discontinued and dexamethasone is administered at a dose of 8 mg every 8 h, with a gradual dose reduction over 4 days after stabilisation. For severe cases, tocilizumab, an IL-6 antagonist that has been shown to be effective in regulating the inflammatory response, is sometimes used. Importantly, due to the short half-life of blinatumomab (about 2 h), discontinuation of treatment quickly reduces symptoms [19]. Of the 26 patients with dexamethasone pretreatment, half did not display CRS, and the other 13 patients had CRS grade 2 or 3 [67].

Infectious complications are also a concern [67,71,72,73], as blinatumomab leads to the destruction of CD19-positive B cells, which leads to hypogammaglobulinaemia and an increased risk of catheter-related bloodstream infections. Compared to standard chemotherapy, blinatumomab has a more favourable infection profile, with a lower incidence of severe infections. For prevention, it is important to follow the rules of asepsis when working with the catheter, and in cases of prolonged hypogammaglobulinaemia, immunoglobulin replacement therapy is considered. In addition, blinatumomab can cause neutropenia, but in cases of moderate to severe infection, granulocyte colony-stimulating factor is recommended to reduce the risk of infection and avoid discontinuation of therapy [19].

Other less common but potentially serious side effects include heart damage associated with cytokine dysfunction, which can lead to heart failure, and haemophagocytic lymphohistiocytosis, which occurs as a complication of a CRS. Bone marrow necrosis is a rare complication that can be caused by microvascular damage, thrombosis, or toxicity [19,67,71]. All of these conditions require timely diagnosis and treatment, as well as consultation with the relevant specialists.

**Figure 6:**
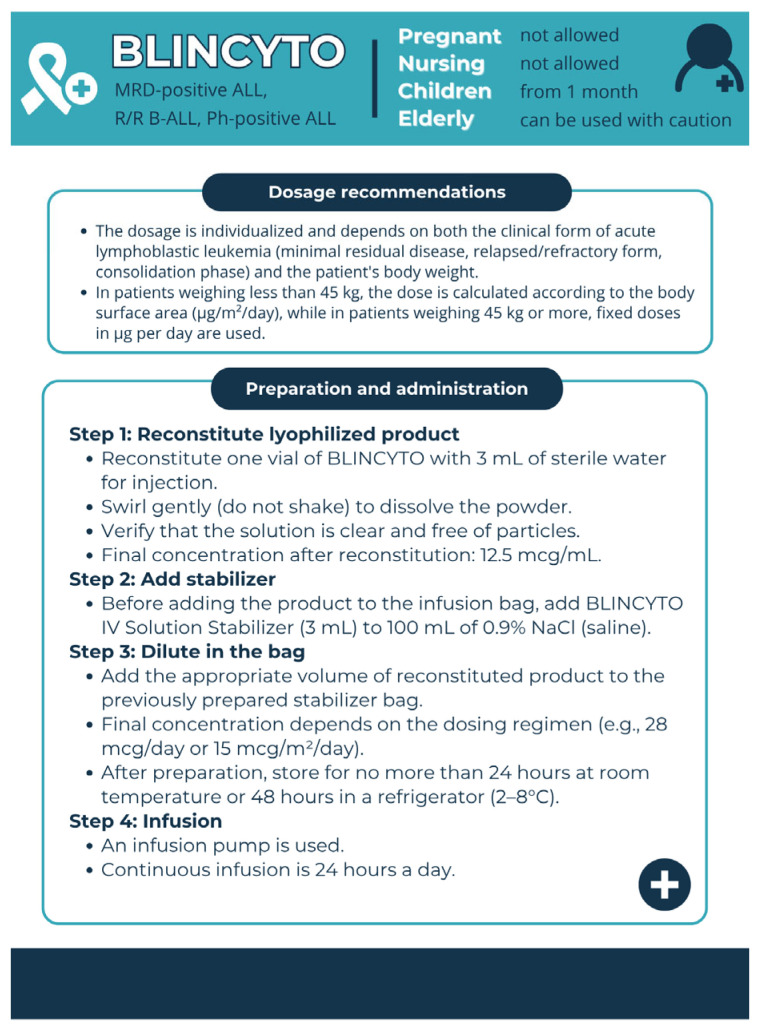
Pharmaceutical aspects of Blincyto (blinatumomab). Data from the FDA website [23] were used.

Pharmaceutical aspects: According to the FDA, blinatumomab must be administered by continuous infusion using a special pump (Figure 6), which complicates the process of using the drug. Due to the lengthy infusion and the complexity of the technical process, patients often face the risk of developing infectious complications [23].

The high cost of medicines reduces their accessibility, especially in low- and middle-income countries. According to pharmacoeconomic studies, it can be assumed that blinatumomab therapy is cost-effective compared to high-risk consolidation chemotherapy (HC3). These results have been confirmed by authors from various countries, such as France [74], Japan [75], and Mexico [76].

One way to reduce costs is through a personalised approach to therapy, which includes blinatumomab, as well as other mAb drugs, for the hospital group. In view of this, there are quality risks in the manufacture of infusion solutions. Pharmacists are currently conducting research to minimise these risks and reduce API losses during manufacturing [77,78,79].

The possibility of subcutaneous administration opens up prospects for simplifying the independent use of blinatumomab [80,81].

The authors of one study [82] have demonstrated (in the low number of patients included) the feasibility of a pharmacist-driven home infusion pathway for blinatumomab and highlight the substantial impact on inpatient drug cost savings for planned short-term inpatient monitoring of blinatumomab recipients [82].

Another team [83] confirms the feasibility of a home-based continuous blinatumomab infusion without adverse effects on safety and propose outpatient protocol leading to cost savings associated with reduced length of stay and an overall improved quality of life for paediatric patients able to receive therapy at home with their caregivers. Special devices (infusion pumps) are provided to patients for independent home use, and additional filters are developed to prevent leakage of the drug [84,85].

#### 3.2.2. Anti-CD22 Therapy for B-ALL

Inotuzumab ozogamicin is a CD22-directed antibody and cytotoxic-drug conjugate (ADC) consisting of three components:

(1) The recombinant humanised immunoglobulin class G subtype 4 (IgG4) kappa antibody inotuzumab, specific to human CD22;
(2) N-acetyl-gamma-calicheamicin, which causes double-stranded DNA breaks;
(3) An acid-cleavable linker composed of the condensation product of 4-(4′-acetylphenoxy)-butanoic acid and 3-methyl-3-mercaptobutane hydrazide that covalently attaches N-acetyl-gamma-calicheamicin to inotuzumab [86].

The average number of calicheamicin derivative molecules conjugated to each inotuzumab molecule is approximately six, with a distribution from two to eight. InO is produced by chemical conjugation of the antibody and small molecule components [86].

**Figure 7:**
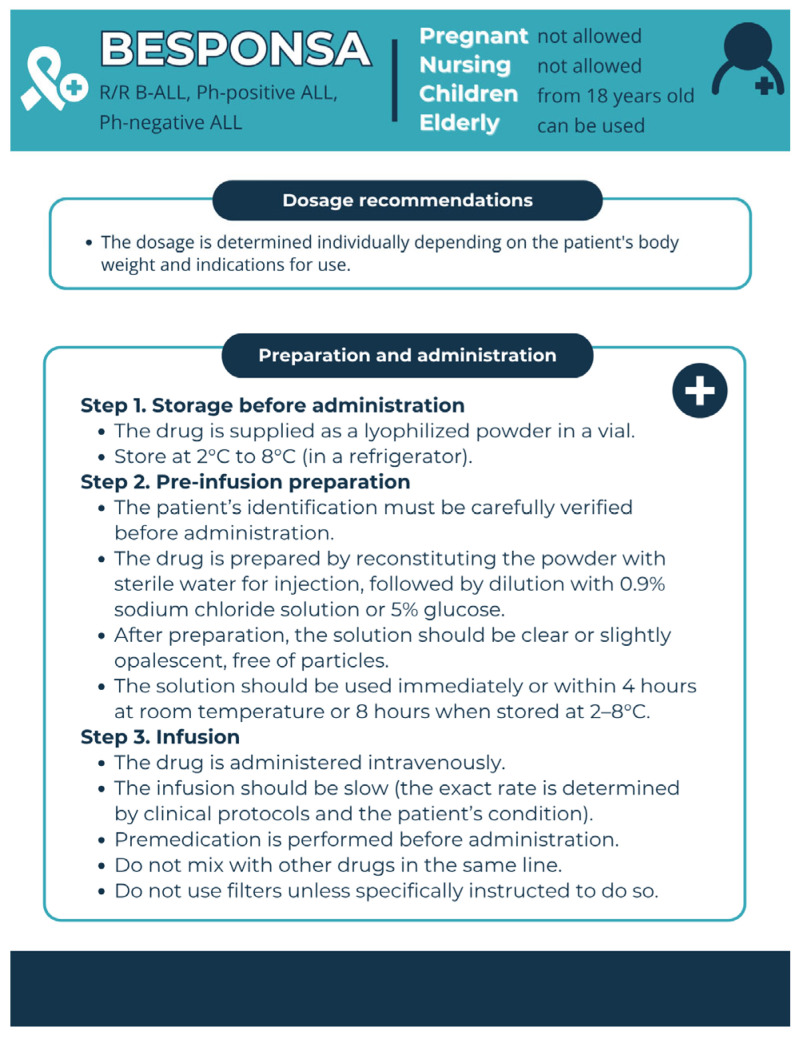
Pharmaceutical aspects of Besponsa (InO). Data from the FDA website [24] was used.

Mechanism of action: InO is a conjugate of a mAb to CD22 (a transmembrane inhibitory receptor specific to B cells, which is an important target for therapy) with a cytotoxic agent (calicheamicin) (Figure 7), which was originally developed for the treatment of B-cell lymphomas but later demonstrated high clinical efficacy in the treatment of ALL, especially in refractory or relapsed cases [70,87,88]. After specific binding of InO to CD22 on the surface of B cells, the drug is rapidly internalised into the cell. Inside the cell, calicheamicin is separated and binds to the DNA small furrow, causing double-stranded DNA breaks. This leads to the induction of leukaemic cell apoptosis. This mechanism provides selective toxicity to CD22-positive B cells, making InO effective in the treatment of B-ALL. It has demonstrated high efficacy in the treatment of ALL, regardless of the level of bone marrow involvement, extramedullary involvement, CD22 expression, and Ph status. Treatment with InO increases the likelihood of allogeneic haematopoietic stem cell transplantation, which is an important factor in improving patient survival [89]. According to the FDA [90], the drug has a complex administration regimen that includes infusions with a gradual increase in dosage to minimise toxic effects.

Side effects: At the same time, InO has a number of side effects that require careful monitoring and timely treatment [70,91,92,93]. One of the most serious is *hepatotoxicity*, in particular, sinusoidal liver obstruction syndrome, which is manifested by elevated bilirubin and transaminase levels, hepatomegaly, right upper abdominal pain, and ascites. This syndrome is potentially fatal and most often occurs after allogeneic stem cell transplantation.

The risks of developing the syndrome increase with the number of InO cycles, the use of alkylators in pre-transplant conditioning, the patient’s age, and elevated bilirubin before transplantation. To reduce the risk, it is recommended to limit the number of InO cycles to two, avoid the use of certain alkylators (thiotepa, melphalan), use ursodiol prophylaxis, and avoid additional hepatotoxic therapy. Early detection of symptoms and monitoring of liver function are key, and in case of pathological developments, supportive therapy with fluid control, diuretics, oxygen, and, if necessary, surgical interventions are indicated [19,92,94].

Another common group of side effects is haematological toxicity, which is manifested by neutropenia, thrombocytopenia, and an increased risk of infections [92,94,95]. Although the incidence of neutropenia and thrombocytopenia with InO is significant, febrile neutropenia is less common compared to standard chemotherapy. To control haematological toxicity, regular monitoring of complete blood counts before each treatment cycle, the use of granulocyte colony-stimulating factor in case of low neutrophil counts, and possible reduction or temporary discontinuation of the drug in cases of severe cytopenia or infections are recommended [19,92].

Infectious complications are associated with a decrease in the number of CD22-positive B cells after therapy, which can increase susceptibility to infections. However, the incidence of infections, such as sepsis and pneumonia, with InO treatment does not exceed the level of standard therapy. Before starting treatment, it is necessary to screen for hepatitis B and provide antiviral prophylaxis for patients with HBsAg-positive or anti-HBc-positive, as well as to individually assess the need for antibacterial and antifungal prophylaxis depending on comorbidities [19,70,91,92].

During infusion, reactions of varying severity may occur, ranging from mild allergic manifestations to severe, life-threatening reactions. Premedication with steroids, antipyretics, and antihistamines is recommended to reduce risk. In the event of severe reactions, the infusion should be stopped immediately, and appropriate treatment should be provided [19,87,92].

Tumour lysis syndrome (TLS) is another potentially dangerous complication that can occur with a high tumour burden at the beginning of treatment. To prevent this syndrome, it is recommended to use cytoreduction with hydroxyurea or cyclophosphamide before the first dose of InO, as well as to use rasburicase, hydration, and allopurinol [19,91,92,94].

Sometimes there is a prolongation of the QT interval on the ECG, which increases the risk of arrhythmias. To minimise this risk, it is recommended to conduct regular monitoring of ECG and electrolytes, avoid concomitant use of other drugs that prolong QT, and monitor symptoms associated with cardiac arrhythmias [19,92].

Thus, the successful use of InO requires a comprehensive approach to monitoring and managing side effects, including regular monitoring of liver function, blood counts, symptoms of infections, and heart rate, as well as the timely use of preventive and therapeutic measures to reduce the risk of complications and improve treatment safety [19,92].

Pharmaceutical aspects (Figure 7): Administration of InO does not require hospitalisation and is appropriate for outpatient use [96,97]. Article [96] included rationale for inpatient InO administration, hospital admission reason, number of InO doses and number of vials used, length of stay, in-hospital mortality, percentage of admissions that were new-starts, outpatient continuation of InO, use of concomitant regimens, and CD22 positivity. Based on the results of this evaluation, appropriate inpatient use guidelines for InO were developed. It was shown that inpatient use of InO was associated with a prolonged length-of-stay and 17% in-hospital mortality and represents a significant cost burden to the health system [96].

Synergism: Synergism with Venetoclax and dexamethasone was discovered as a base for combination therapy prospects [88].

Pharmacoeconomics: Clinical- and cost-effectiveness evidence for inotuzumab was studied as part of National Institute’s for Health and Care Excellence (NICE) single technology appraisal process. The Appraisal Committee concluded that the ICER for inotuzumab was within the range usually considered cost effective (for end-of-life care) and recommended inotuzumab within its licenced indication [98]. Inotuzumab has been presented as a cost-effective option in the treatment of ALL in other countries, such as the USA [99], Bulgaria [100], Norway and Sweden [101], and Taiwan [102]. In the first R/R setting, patients who used InO had significantly lower all-cause and ALL-related costs compared with patients who used Blina, in part driven by hospitalisation patterns [103].

### 3.3. Targets of Immunobiological Therapy for AML

AML is a cancer of bone marrow stem cells that often leads to death despite available treatments. AML is diagnosed predominantly at an older age (median age at diagnosis 68 years) [104] and has an estimated 5-year overall survival rate of 32% (up to 50% in younger patients and less than 10% in patients over 60 years of age) [105].

The latest drugs are an important achievement, especially for patients with subtypes that are difficult to treat with classical therapy [106]. The main classes of immunotherapeutic drugs for the treatment of AML include conjugated antibodies that combine the specificity of antibodies with powerful cytotoxins or radioactive isotopes to selectively kill leukaemic cells and BsAbs and simultaneously bind to leukaemia cells and T cells to activate the immune response, and checkpoint inhibitors that block immunosuppressive pathways, release the immune brake, and stimulate the anti-tumour function of T cells [38]. Currently, the only approved immunobiological drug for the treatment of AML is GO, while the rest are in clinical trials and are being actively investigated.

Conjugated mAbs are a promising therapeutic approach to AML, offering targeted delivery of cytotoxic agents to malignant cells [107].

Gemtuzumab ozogamicin is an antibody–drug conjugate composed of the CD33-directed mAb (hP67.6; recombinant humanised immunoglobulin [Ig] G4, kappa antibody produced by mammalian cell culture in NS0 cells) that is covalently linked to the cytotoxic agent N-acetyl gamma calicheamicin [108].

GO contains both conjugated and unconjugated gemtuzumab. The number of conjugated calicheamicin derivatives per gemtuzumab molecule ranges from predominantly 0 to 6, with an average of 2 to 3 moles of calicheamicin derivative per mole of gemtuzumab. CD33 is a transmembrane receptor expressed on myeloid cells and AML blasts, but not on normal haematopoietic stem cells, making it a suitable target for antibody–drug conjugates [109].

**Figure 8:**
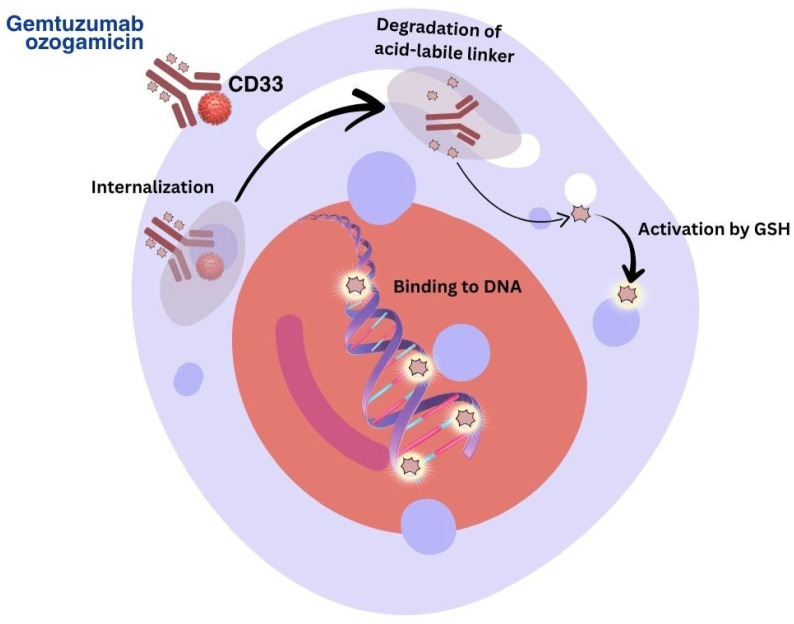
Mechanism of action of GO.

Mechanism of action: GO is a conjugate consisting of a mAb targeting CD33 (Figure 8) linked to a cytotoxic calicheamicin derivative [110]. The mechanism of action of the drug is associated with an acid–labile linker that releases the toxin in lysosomes, inducing DNA damage and apoptosis [111].

Early Phase I studies in relapsed/refractory AML showed moderate clinical activity, leading to accelerated FDA approval in 2000 for CD33-positive AML in the elderly. However, due to limited efficacy in the subsequent SWOG S0106 trial and concerns about toxicity, particularly veno-occlusive disease (VOD), the drug was withdrawn in 2010. Later clinical trials, such as the ALFA-701 study, showed that fractionated lower doses (3 mg/m^2^ on days 1, 4, and 7) improved safety while maintaining efficacy. This regimen reduces peak serum concentrations and the risk of adverse effects, which is especially important in combination with intensive chemotherapy [111]. Re-approval by the FDA was supported by these newer data and an improved understanding of its pharmacokinetics and toxicity profile in 2017 for newly diagnosed and relapsed/refractory AML [112].

Side effects: The main side effects are stated as hepatotoxicity, including VOD, haemorrhage, and infusion-related reactions [105,113,114].

As mentioned above, the most common complication of GO therapy is VOD. This is a serious, potentially life-threatening complication that occurs as a result of endothelial damage induced by toxic metabolites during conditioning. It is diagnosed on the basis of symptoms such as weight gain, hepatomegaly, ascites, and jaundice. Therapy with GO has been linked to VOD via the effect of calicheamicin on CD33+ sinusoidal endothelial cells [113,114,115]. Clinical trials have reported varying incidences of VOD: from 3% at lower doses to 28% when combined with thioguanine, and 15% at a dose of 9 mg/m^2^. Recent evidence suggests that the risk of VOD is lower than originally thought, likely due to lower or fractionated dosing and better risk surveillance [113].

Nevertheless, VOD remains a life-threatening complication that requires close monitoring. Preventive measures are recommended, such as delaying haematopoietic stem cell transplantation ≥ 3 months after drug use and using ursodeoxycholic acid in high-risk patients. Signs of hepatotoxicity and liver function parameters should be carefully monitored, especially if azoles are used. Transient elastography is being investigated for the early detection of VOD. Treatment should be initiated immediately if suspected, starting with fluid balance correction and diuretics. Defibrotide remains the only approved treatment for VOD, due to its endothelioprotective properties and proven efficacy, especially in cases of haematopoietic stem cell transplantation after therapy [113].

In the ALFA-0701 study, persistent grade 3/4 thrombocytopenia was significantly more common in the GO group (16%) than in the control group (3%), and the term “Complete remission with incomplete platelet recovery” was coined to describe the frequent absence of complete platelet count recovery among treatment-responders. Patients receiving the drug required more frequent platelet transfusions after each course of treatment. To treat thrombocytopenia in patients, regular monitoring of complete blood counts (usually 2–3 times a week) and clinical observation are important. GO should be postponed or discontinued in cases of severe bleeding and persistent thrombocytopenia, and supportive care should be provided. In the ALFA-0701 protocol, the drug was contraindicated during consolidation if the platelet count remained <100 × 10^9^/L by day 45 after the start of chemotherapy [113].

Infusion-related reactions, typical of mAbs, are usually mild (e.g., fever, chills, hypotension, respiratory symptoms) and more likely during the first infusion. The drug should not be administered to patients with hypersensitivity to its components; premedication with corticosteroids, antihistamines, and acetaminophen one hour before administration, as well as close monitoring of vital signs, is recommended. Severe or life-threatening infusion reactions require permanent discontinuation of GO [113].

*TLS* is another potential complication that can lead to metabolic disorders that cause renal dysfunction, arrhythmias, seizures, or even death. High-risk patients (e.g., white blood cell count ≥ 30,000/μL) should undergo leukoreduction with leukapheresis or hydroxyurea before administration, with adjustments to induction regimens as necessary. Preventive measures, such as hydration and antihyperuricemics (e.g., allopurinol), should be used. If the syndrome develops, aggressive treatment, including electrolyte correction and rasburicase, is necessary to prevent complications [113]. In combination with standard chemotherapy a slightly higher rate of severe gastrointestinal (GI) toxicity was reported [116].

**Figure 9:**
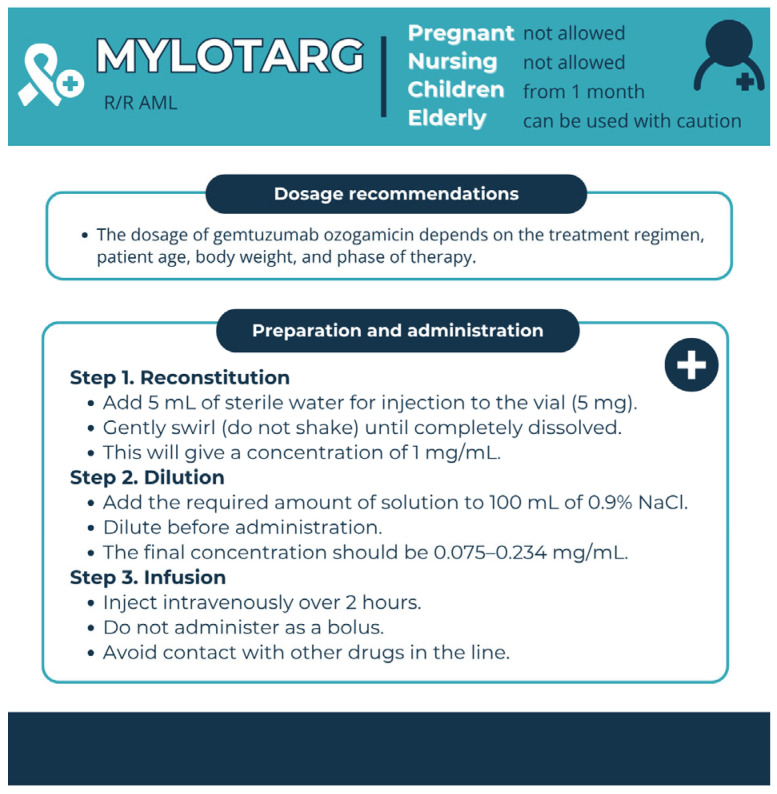
Pharmaceutical aspects of Mylotarg (GO). Data from the FDA website was used [117].

Pharmaceutical aspects are summarised in Figure 9.

Pharmacoeconomics. Results of cost-effectiveness analysis indicate that GO in combination with SOC is a cost-effective first-line treatment option for adult patients with de novo AML from the perspective of the healthcare payer in the UK [118], the US [119], Spain [120], Italy [121], etc.

### 3.4. Targets of Immunobiological Therapy for CLL

CLL is the most common type of leukaemia. It usually occurs in elderly patients and has a highly variable clinical course. Leukaemic transformation is initiated by specific genomic changes that interfere with the regulation of proliferation and apoptosis in clonal B cells [122].

**Figure 10:**
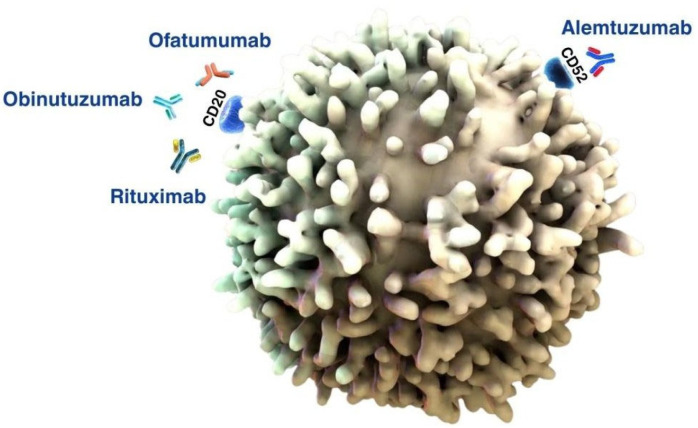
Targets of mAb drugs on B cells in CLL.

To date, the main molecular targets for approved mAbs in the treatment of CLL are CD20 and CD52 (Figure 10). These are surface antigens expressed on B-lymphocytes; targeting allows for the selective destruction of tumour cells with minimal impact on other tissues.

#### 3.4.1. Anti-CD20 Therapy for CLL

The first class of antibodies used to treat CLL are mAbs against CD20, including rituximab, ofatumumab, and obinutuzumab. These agents have revolutionised the treatment of CLL by effectively targeting B cells through the CD20 antigen [123] (Figure 10).

CD20 is a surface glycoprotein expressed on mature B cells, and its expression is restricted to the B-cell lineage. Immature haematopoietic stem cells and most haematopoietic cells do not express it; therefore, mAbs to CD20, such as rituximab, ofatumumab, and obinutuzumab, have been developed and are used to treat mature B-cell malignancies. Anti-CD20 antibodies are classified into two groups, type I and type II, based on differences in epitope and binding mode. Rituximab and ofatumumab belong to type I. Such antibodies can stabilise CD20 molecules on lipid rafts, which leads to increased C1q binding and induction of strong complement-dependent cytotoxicity. In contrast, the type II antibody obinutuzumab cannot stabilise CD20 on lipid rafts, resulting in a reduced binding potential to C1q and lower levels of complement-dependent cytotoxicity. However, they can directly cause cell death [124].

Rituximab, a chimeric IgG1 mAbs directed against CD20, was the first FDA-approved mAb for cancer treatment and has had a significant impact on the treatment of B-cell malignancies, including CLL. Rituximab is not currently used as widely in CLL, as newer drugs are available. Current studies suggest that it may be used experimentally in CD20-positive B-ALL. This section provides information about rituximab to provide an overview of its historical role in the development of therapy, as well as a general understanding of its mechanism of action and pharmacological properties.

Mechanism of action: Rituximab kills B cells through complement-dependent cytotoxicity, antibody-dependent cell-mediated cytotoxicity, and induction of apoptosis. Although it was originally developed for follicular lymphoma due to its high CD20 expression, its efficacy in CLL is reduced due to its lower antigen density and high tumour burden, which results in lower plasma concentrations of rituximab and faster clearance. Early studies of monotherapy in CLL showed moderate response rates, with improved results with more frequent or higher dosing regimens. However, responses were mostly partial and short-lived, prompting a shift to combination therapy. Despite the variability of patient response due to pharmacokinetics and CD20 expression, rituximab remains the standard therapy for CLL, especially in combination with chemotherapy [125].

Side effects: The most common and serious side effects of the drug are associated with infusion reactions. In randomised controlled trials, infusion-related reactions were observed in 80–90% of patients receiving rituximab. They usually occur within 30–120 min after the first infusion and range from mild to life-threatening. Reactions to the infusion may include fever, chills, skin rash, urticaria, angioedema, hypotension, ventricular fibrillation, shock, anaphylaxis, and death [126]. Thirty minutes before the infusion, patients are premedicated with analgesics/antipyretics (e.g., acetaminophen), antihistamines (e.g., diphenhydramine), and steroid hormones (e.g., methylprednisolone).

A urethrostatic agent (e.g., allopurinol) and aggressive hydration are often used before rituximab for the treatment of high burden tumours [127].

Rituximab should be used with caution in elderly patients and patients with cardiopulmonary disease. Patients over 55 years of age have a higher incidence of serious complications. Rituximab should also be avoided in anyone with a severe infection [126]. The drug is classified as a category C drug by the FDA: its use during pregnancy is possible only when the expected benefit to the mother outweighs the potential risk to the foetus. Transient B-cell lymphocyte depletion has been reported in infants exposed in utero to rituximab. Although rituximab is secreted into the breast milk of lactating monkeys, there are insufficient data for or against its use during breastfeeding in humans [127].

Rituximab treatment is associated with an increased risk of infections, especially during the first year after therapy. Among 356 patients with lymphoid malignancies, 31% developed bacterial, 10% viral, and 1% fungal infections, and most of them were without serious complications. Patients with rheumatoid arthritis treated with rituximab with methotrexate had a higher rate of serious infections compared to placebo. Infections were often controlled, although rare severe outcomes such as toe amputation or deaths associated with sepsis occurred [128]. Serious viral infections, including hepatitis B virus reactivation, have also been reported following rituximab use, which can be fatal and require treatment discontinuation. Screening is important before starting rituximab treatment, as studies show up to 60% reactivation in HBV-positive patients, with high rates of severe hepatitis and death [129]. Evidence for the safety of rituximab in latent hepatitis C infection remains insufficient [126].

During therapy, it is also important to monitor diuresis and serum creatinine trends due to the risk of developing acute TLS. TLS occurs when the rapid destruction of malignant cells releases intracellular contents, including uric acid, which can crystallise in the renal tubules and lead to acute kidney injury. Early signs of TLS include hyperkalaemia, hypocalcaemia, hyperphosphaemia and hyperuricaemia. Prevention with intravenous hydration and antihyperuricemic agents such as allopurinol is recommended before infusion [126]. Although urine alkalinisation remains controversial, patients with high leukocyte counts, Burkitt’s lymphoma, or renal impairment may benefit from rasburicase to reduce uric acid levels [130]. Treatment of TLS is predominantly supportive, requiring close monitoring of electrolytes, renal function, and fluid balance, and dialysis if necessary [131]. Renal function should also be closely monitored when rituximab is used in combination with cisplatin.

Cardiovascular arrhythmias have been reported in patients with rheumatoid arthritis treated with rituximab. Although rare, cases of myocardial infarction, severe hypertension, cardiac tamponade, and heart failure with fatalities have been documented after therapy, even in patients without previous heart disease [126]. The mechanism of myocardial infarction caused by rituximab may be the release of cytokines after B-cell death, which leads to platelet activation, vasoconstriction, and plaque rupture. The risk may be higher in patients with pre-existing atherosclerosis and vulnerable plaques, as well as those with advanced malignancies. Adequate hydration before starting rituximab chemotherapy (especially at the first dose of infusion) may be a preventive strategy [132]. In addition, in the presence of thrombotic thrombocytopenic purpura, a new cardiogenic shock was reported, which disappeared after discontinuation of rituximab. Also, a fatal case of myocarditis was reported in a patient with follicular lymphoma, which was confirmed at autopsy [126]. To prevent cardiogenic shock, blood pressure and cardiac output should be carefully monitored, especially during rituximab infusion. The patient’s history and risk factors should be assessed before starting therapy. Diuresis and signs of target organ perfusion should be regularly monitored. To prevent acute coronary syndromes, a baseline ECG and 2D echocardiogram should be performed before starting therapy in at risk patients. If symptoms develop, cardiac enzymes should be monitored. Aspirin/statins/β-blockers should be prescribed to patients with pre-existing coronary heart disease unless contraindicated, and the concomitant use of drugs that may cause coronary vasospasm or increase cardiac demand should be avoided. It is also important to avoid electrolyte imbalances (especially K^+^, Mg^2+^), which contribute to the development of arrhythmias [133].

In 2011, a review of 418 patients treated with rituximab monotherapy reported that 5.3% developed pulmonary side effects, approximately two-thirds of which were infectious, and almost a quarter progressed to interstitial lung disease (ILD). Although the ILD improved in most cases, only half of these patients received intravenous corticosteroids. Hypoalbuminemia was identified as an independent risk factor for pulmonary complications. Rare but serious pulmonary toxicities associated with rituximab included asthmatic status, bronchiolitis obliterans, hypersensitivity pneumonitis, and diffuse alveolar haemorrhage. Bronchiolitis obliterans and hypersensitivity pneumonitis showed improvement after the discontinuation of rituximab and steroid therapy, whereas the response in cases of diffuse alveolar haemorrhage was inconsistent [126].

Patients who develop new neurological signs while taking rituximab require neuroimaging and lumbar puncture unless another cause is identified [126]. The known cases include ischaemic stroke, seizures, epilepsy, and serotonin syndrome [134]. Progressive multifocal leukaemia (PML) due to the reactivation of JC virus is often observed in patients with prior immunosuppression or stem cell transplantation. The detection of JC virus in the cerebrospinal fluid confirms PML, which is an indication for the discontinuation of rituximab [126].

Skin side effects are relatively common. In one study involving 356 patients, 37% developed rash, pruritus, or urticaria, and 2% had serious dermatological reactions [135]. Serious skin reactions associated with rituximab include paraneoplastic vesicles, lichenoid and vesiculobullous dermatitis, Stevens–Johnson syndrome, and toxic epidermal necrolysis [136]. These reactions can occur up to 3 months after infusion and require the discontinuation of rituximab [126].

**Figure 11:**
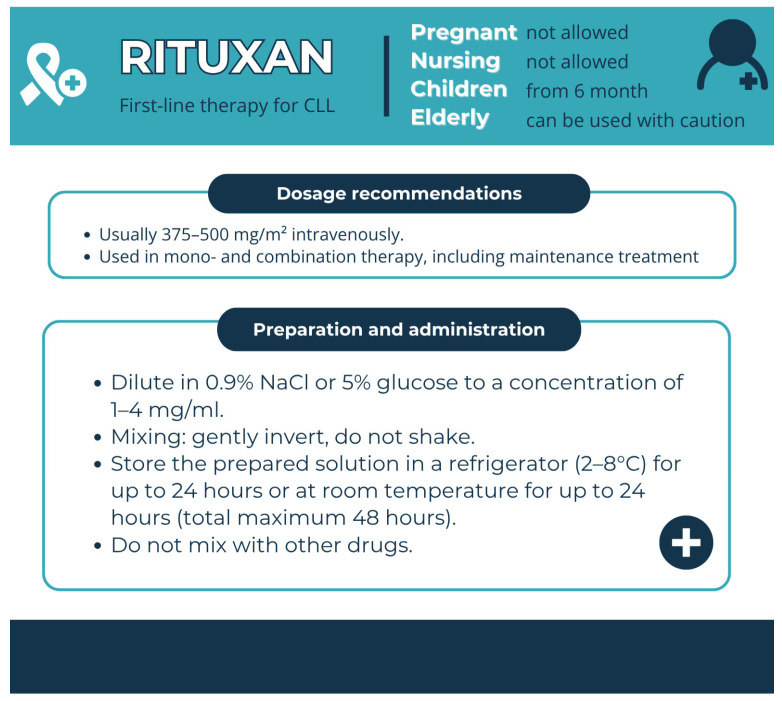
Pharmaceutical aspects of Rituxan (rituximab). Data from the FDA website [29].

Pharmaceutical aspects: Rituximab is administered as an intravenous infusion. For patients who are unresponsive, it is started at 50 mg/h and increased by 50 mg/h every 30 min to a maximum of 400 mg/h (Figure 11).

Biosimilars: Due to patent expiration, many companies have elaborated biosimilars of rituximab. Among them are those that were created in the US and EU, for example, Rixaton, Mabthera, Truxima, Riabni, Ruxience, etc. Comparable clinical efficacy and safety of the reference rituximab and its biosimilars has been established in randomised trials [137,138,139,140]. The similarity in long-term efficacy and safety of biosimilar rituximab to the original was confirmed by combining direct evidence with visual examinations [141].

Pharmacoeconomics: Cost-effectiveness of radiotherapy and rituximab was comparable to that of other treatments for advanced-stage follicular lymphoma, which replaced an older standard of care [142]. It was the introduction of biosimilars that made it possible to significantly increase the availability and cost-effectiveness of rituximab. In particular, the positive impact on budget savings in the healthcare systems of Brazil [143], Italy [144], and 28 European countries [145] has been confirmed.

The second drug is ofatumumab, which is a fully human mAb (IgG1) that binds to a unique epitope on the human CD20 molecule and is expressed on the surface of B cells.

Mechanism of action: It specifically binds to both small and large extracellular loops of the CD20 molecule. The Phase I-II study demonstrated that ofatumumab was well-tolerated and resulted in objective responses. In the Phase II study of ofatumumab, the response rates were 58% and 47%, respectively. This study led to accelerated approval of the drug by the FDA for the treatment of CLL refractory to fludarabine and alemtuzumab. The higher dose of ofatumumab resulted in a higher complete response rate (50%) compared to the lower dose group (32%) (overall response rate, 77% and 73%, respectively) [146].

Side effects: Ofatumumab was well-tolerated, with almost all adverse events being grade 1/2, demonstrating similar toxicity to that expected in the same patient population as rituximab. Of the adverse events, 56% were infusion-related, and they generally decreased in number and severity with subsequent doses. At the same time, 51% of patients experienced infections, and 15% experienced haematological toxicity. The overall response rate was 50%. The response time was fast: 62% of patients responded within 4 weeks [147].

Other common complications included neutropenia (48%), nausea (41%), infections (38%), thrombocytopenia (26%), rash (25%), vomiting (23%), fever (21%), headache (18%), and fatigue (18%) [147].

In patients with previously untreated CLL, the recommended dose and regimen of ofatumumab is 300 mg on day 1, followed by 1000 mg on day 8 of the first 28-day cycle, and then 1000 mg on day 1 of subsequent 28-day cycles. Treatment should be administered for a minimum of 3 cycles, until the best response is achieved or a maximum of 12 cycles is reached. In a Phase III study of ofatumumab in combination with chlorambucil, the oral drug was administered at a dose of 10 mg/m^2^ on days 1–7 of a 28-day cycle [148].

Patients receiving ofatumumab should be pretreated with acetaminophen, antihistamines, and corticosteroids. Infusion facilities should be adequately equipped to monitor and treat infusion reactions [148].

During the first infusion (cycle 1, day 1, dose 300 mg), the rate should be started at 3.6 mg per hour (12 mL per hour). Cycle 1, day 8, and subsequent infusions (cycles 2–12, dose 1000 mg) can be started at 25 mg per hour [148].

If no infusion-related adverse reactions are observed, the infusion rate of ofatumumab may be increased every 30 min. If a grade ≥ 3 infusion-related event is observed during the previous administration of ofatumumab, the initial infusion rate should be reduced to 12 mg per hour [148].

**Figure 12:**
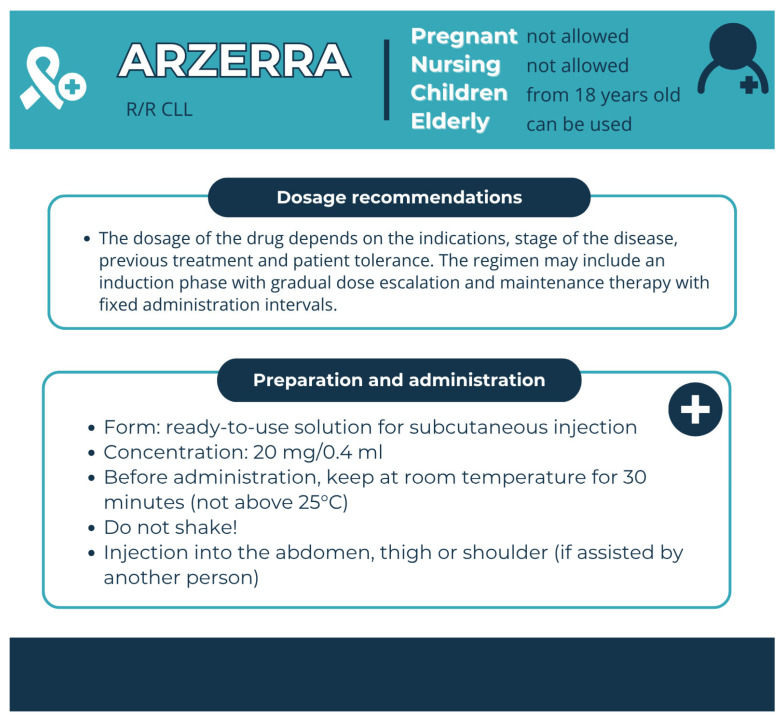
Pharmaceutical aspects of ARZERRA (ofatumumab). Data from the FDA website [26].

Pharmaceutical aspects: Ofatumumab is an API of Arzerra; it is administered as an intravenous infusion (Figure 12).

Pharmacoeconomics. Arzerra is now not available for commercial purchase. Novartis provides the drug directly, at no cost to patients, on a reimbursement programme. The cost-effectiveness of this medicinal product has previously been proven in different countries [149,150,151].

CLL is characterised by a constant relapse rate and increasing resistance to therapy [152]. Obinutuzumab is the second new-generation anti-CD20 drug (after ofatumumab) to enter clinical practice in CLL. Its superiority in combination with chlorambucil and venetoclax led to its approval as a first-line drug [153].

Mechanism of action: Type I antibodies bind to CD20 and cause a rapid redistribution of the antibody–antigen complex into a lipid raft. This complex results in only weak direct cell death or apoptosis, but strong complement-dependent cytotoxicity via recruitment of C1q. Ofatumumab has a particularly high affinity and potent complement-dependent cytotoxicity activity due to a distinct binding site in the transmembrane protein CD20, which is different from the binding site of rituximab [154]. In contrast, type II antibodies, such as obinutuzumab, do not localise the antibody–antigen complex to lipid rafts and therefore induce only very weak complement-dependent cytotoxicity, which is 10–100 times weaker than with rituximab or ofatumumab [155].

However, the reduced FcγRIIb-mediated internalisation of CD20 increases the ability to bind and activate natural killer cells and the subsequent immune effector function. In addition, obinutuzumab induces cell death by homotypic aggregation, which leads to the aggregation of malignant B cells by antibodies and subsequent non-apoptotic cell death without the involvement of immune effector cells. It has been suggested that binding of obinutuzumab leads to activation of the family of rokinases involved in B-cell receptor activation, as well as cytoskeletal rearrangement. Preclinical observations confirmed that obinutuzumab induced rapid relocalisation of actin filaments, together with cell surface antigens, to cell junctions, and thereby also activated lysosomes, which play an important role in triggering caspase-independent death. In particular, it has been shown that when type II antibodies bind to CLL cells, lysosomes release various enzymes, including cathepsin B, and thereby induce cell death independently of caspases and without the involvement of B-cell lymphoma. This mechanism is not fully understood but has been previously described for other antigens and is of particular interest to patients with CLL, who often have immune disorders. In addition, obinutuzumab mediates increased NK cell binding and activation, especially through FcγRIIIa, which ultimately leads to increased antibody-dependent cell-mediated cytotoxicity and antibody-dependent cellular phagocytosis [156].

Side-effects: Therapy-related adverse events occurred in more than 10% of clinical trial participants (n = 240) receiving obinutuzumab with chlorambucil: infusion reactions (69%; 21% grade 3/4), neutropenia (40%; 34% grade 3/4), thrombocytopenia (15%; 11% grade 3/4), anaemia (12%), and pyrexia and cough (10% each). Laboratory abnormalities (>20%) have also been reported, including hypocalcaemia, hyperkalaemia, hyponatraemia, elevated serum creatinine and liver function tests, and hypoalbuminemia. Infusion reactions are the most common side effects reported with obinutuzumab. Signs and symptoms may include hypotension; tachycardia; and respiratory symptoms such as dyspnoea, wheezing, bronchospasm, throat and laryngeal irritation, and laryngeal oedema. After the first dose, the incidence of infusion reactions is significantly reduced (<3%). In addition, in clinical trials, no grade 3 or 4 infusion reactions occurred after the first full dose. In patients who are not at risk of developing a hypertensive crisis, a pharmacist should consider discontinuing antihypertensive medications 12 h before and during obinutuzumab administration. Due to the risk of thrombocytopenia and haemorrhage, discontinuation of concomitant medications that may increase the risk of bleeding, especially during the first cycle, should be considered. Approximately 5% of patients experienced acute thrombocytopenia within 24 h of infusion, and all fatal haemorrhagic events occurred during the first cycle [157]. Reactions to the first dose of obinutuzumab infusion can be significantly reduced by using chlorambucil to reduce lymphocyte counts before obinutuzumab and by using a very slow initial obinutuzumab infusion rate [158].

Infusion-related reactions occur in about 60% of patients with CLL treated with obinutuzumab, with 10% of them being grade ≥ 3. To reduce the risk of adverse events, premedication (glucocorticoids, acetaminophen, and antihistamines) and slow intravenous infusion are used. The standard infusion of obinutuzumab lasts ≥195 min, which, together with premedication, creates a significant financial burden for patients and infusion centres. In this regard, starting from the second cycle, some institutions use a short-term infusion lasting 90 min, which is considered safe for patients if there were no severe grades (≥3) in the first cycle (Figure 12) [158].

Obinutuzumab has the ability to induce hepatitis B reactivation and PML, which occurs as a result of infection with human polyomavirus type 2. Patients with new onset or changes in pre-existing neurological symptoms should be evaluated immediately, and patients diagnosed with PML should not receive further treatment with obinutuzumab [159].

**Figure 13:**
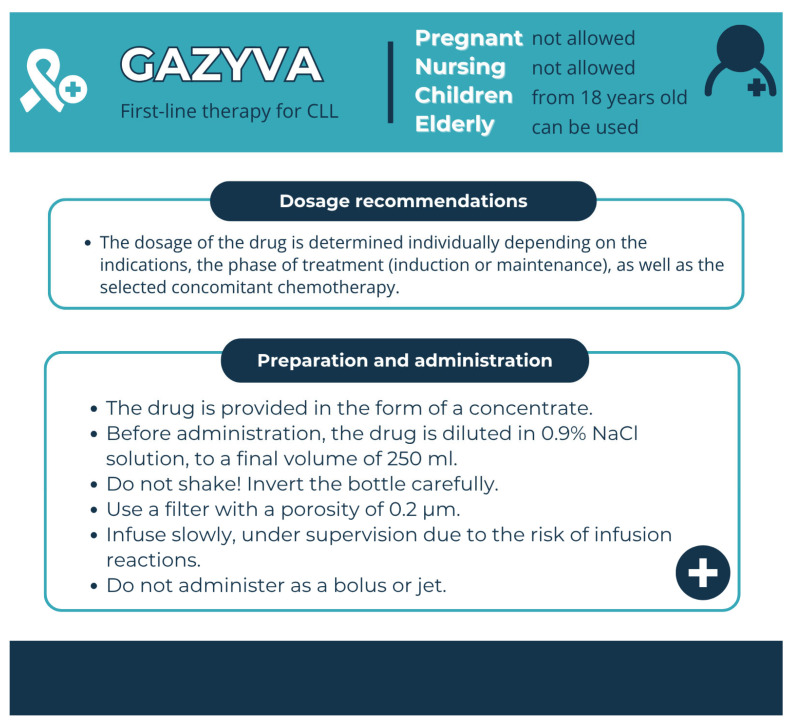
Pharmaceutical aspects of Gazyva (obinutuzumab). Data from the FDA website [27].

Pharmaceutical aspects: Obinutuzumab is an API of Gaziva (US) and Gazyvaro (EU); it is administered as an intravenous infusion (Figure 13).

Pharmacoeconomics: The economic studies conducted in China, the United States, Japan, Italy, and Norway have demonstrated that obinutuzumab-based chemotherapy is cost-effective compared to other chemotherapy. Although obinutuzumab significantly prolonged PFS and was cost-effective, its safety profile was considered to be lower [160]. Obinutuzumab in combination with traditional chemotherapeutics is projected to be cost-effective versus rituximab biosimilars plus chemotherapy in the United States as a first-line treatment for FL, driven by increased QALYs for obinutuzumab combinations and cost savings from delayed disease progression [161]. Cost-effectiveness analysis of venetoclax in combination with obinutuzumab (VenO) could be used to support decision making in both clinical applications and reimbursement of VenO [162].

#### 3.4.2. Anti-CD52 Therapy for CLL

Alemtuzumab is a humanised mAb against CD52 (Figure 10). CD52 is a 21–28 kD cell surface glycopeptide expressed on virtually all human lymphocytes, monocytes, and macrophages, a small subset of granulocytes, but not erythrocytes, platelets, or bone marrow stem cells. CD52 is expressed on all CLL cells and indolent B cells [163].

Mechanism of action: Cross-linking of CD52 on B-cell and T-cell lymphoma cell lines has been shown to inhibit cell proliferation [164]. In CLL cells, alemtuzumab induced apoptosis, complement-dependent cytotoxicity, and antibody-dependent cellular cytotoxicity in vitro. The ubiquitous expression of CD52 on normal lymphocytes and monocytes predicted increased neutropenia, lymphopenia, and infectious complications associated with the clinical use of alemtuzumab. The drug cleared peripheral blood CLL cells in 97% of patients but was less effective in bone marrow (36%) and nodal (7%) disease [165].

Side effects: Due to the ubiquitous expression of CD52 on lymphocytes and monocytes, alemtuzumab causes significantly greater infusion, haematological, and immune toxicity than rituximab, and careful monitoring and prevention of potential infections is required for all administration. Infusion toxicity for IV alemtuzumab decreases with an increased dosing regimen, and infusion toxicity usually decreases with further administration [165]. Corticosteroid administration significantly reduces infusion toxicity, but haematological and infectious complications still exist. However, infectious complications can be treated with adequate antibiotic prophylaxis and careful monitoring of CMV reactivation and other potential infections [166,167].

**Figure 14:**
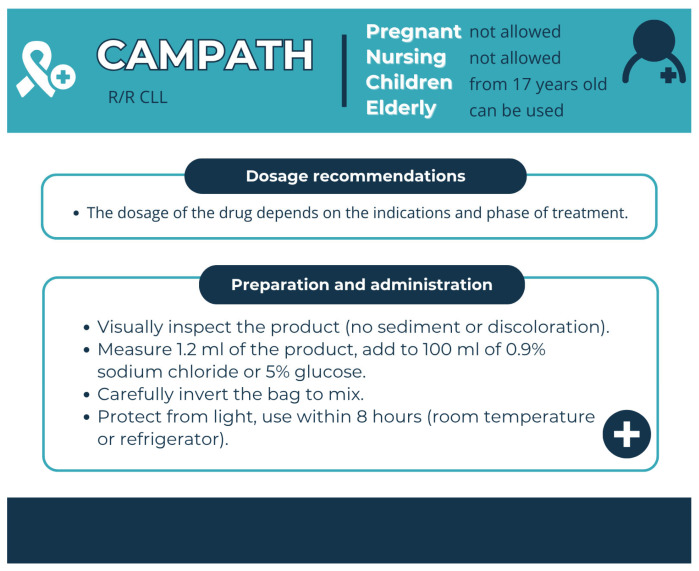
Characteristics of Campath (alemtuzumab). Data from the FDA website [28].

Pharmaceutical aspects: Campath with Alemtuzumab (30 mg/mL) is used as a single agent for treatment of B-CLL, while Lemtrada (10 mg/mL) is indicated for relapsing forms of multiple sclerosis (MS). It is administered as an intravenous infusion (Figure 14).

### 3.5. Chronic Myeloid Leukaemia

CML is BCR-ABL1-positive and is classified as a myeloproliferative neoplasm predominantly composed of proliferating granulocytes with a Ph chromosome/translocation. CML affects both peripheral blood and bone marrow [168].

The annual incidence rate of CML is 0.87 per 100,000, increasing with age to 1.52 in patients over 70 years of age. There is a slight male predominance, and the average age at diagnosis is 56 years [168].

To date, there are no approved mAb drugs for the treatment of CML due to the lower safety of such drugs compared to lymphocytosis. In lymphoid cancer, therapy often targets B-cell markers, such as CD19 or CD20, which are not specific to cancer cells but can be targeted without severe consequences, as the body can tolerate the loss of normal B cells. Instead, most of the known myeloid markers (e.g., CD33, CD123, CLL-1) are common to healthy myeloid progenitor cells. Targeting these markers often leads to collateral damage to normal bone marrow, resulting in significant bone marrow suppression [169,170].

### 3.6. The Role of the Pharmacist in the Treatment of Various Types of Leukaemia with mAbs-Based Drugs

MAbs are usually categorised as high-risk drugs and are subject to specific management regulations [171].

In addition to their high specificity, relatively low toxicity and high efficacy, mAb-based drugs can cause quite serious side effects and have severe consequences for the patient’s health if used incorrectly. Many of these side effects are avoidable and could be reduced with the participation of a pharmacist in the treatment (Table 1). Therefore, we believe that a multidisciplinary approach to treatment is necessary. In this case, we propose that both a physician and a pharmacist should be present during the treatment process, who will jointly provide quality medical and pharmaceutical care to the patient, with the goal of reducing the risk of complications and adverse events from therapy.

When prescribing mAbs drugs, a physician should analyse the patient’s condition, anamnesis, and examination results and select a particular type of drug according to a specific dosage regimen that takes into account the functional state of the body. However, anticancer therapy is quite complex, so safe and effective treatment is impossible without the participation of a pharmacist.

At the stage of drug selection, a pharmacist should check compatibility with concomitant therapy; when using drugs, it is important to assess the correct dosage, dilution and storage conditions; it is also important to monitor premedication. Since one of the main disadvantages of this therapy is the cost of preparations, it is important to ensure individualised dosing. This approach will allow one vial to be distributed among several patients in a hospital setting, while maintaining the storage, use and sterility of the drug, and at the same time reducing the financial burden on a particular patient. At the same time, it is the pharmacist who should control this approach based on the physicochemical properties of the drug. Another important mission of the pharmacist is to provide pharmaceutical education to the patient on how to use the medicine. For example, blinatumomab therapy is partially self-administered, so it is important that the patient understands how to control and maintain the infusion pump, adheres to hygiene rules, and ensures timely and correct dosage. Autoimmune disorders are another serious complication that accompanies any mAbs therapy. A significant proportion of such side effects are caused by incorrect administration of drugs or violation of the preparation, dilution, or infusion regimen, which causes contamination and infection of the patient. In the absence of an integrated approach and coordinated cooperation between a doctor, nurse, and pharmacist, the risks of complications increase significantly. That is why pharmaceutical care in immunotherapy should become a mandatory component of modern oncology practice. Such specialists not only prevent potentially dangerous mistakes but also optimise therapy, improving clinical outcomes and the quality of life of patients [172].

MAb drugs not only possess the biological toxicity of chemical drugs but also exhibit the low-temperature preservation characteristics of cold chain drugs. These risks are present throughout drug reception, storage, order auditing, preparation, compounding, delivery, and collection and analysis of ADRs [171].

In addition to the general risks associated with the manufacture of infusion solutions, pharmacists must take into account the ability of mAbs to aggregate and degrade when selecting technologies and excipients [173].

Important aspects related to packaging, storage and shelf life of medicinal products are summarised in Table 2 and Table 3.

## 4. Conclusions

**Figure 15:**
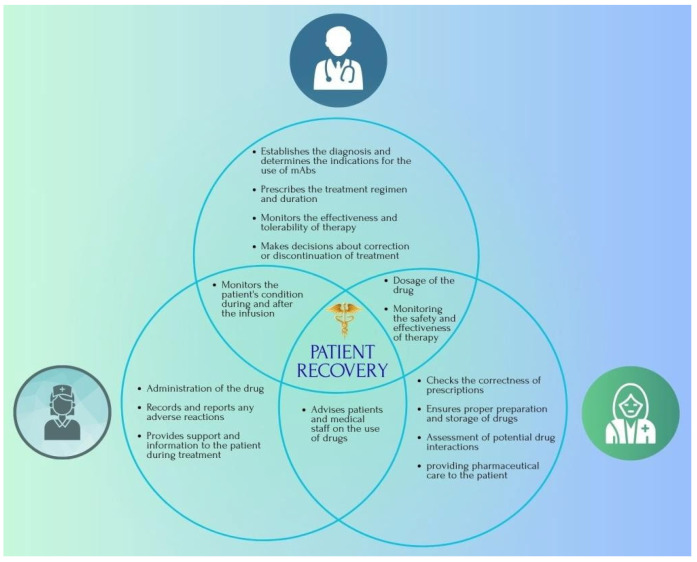
Multidisciplinary approach to mAbs therapy.

MAb-based drugs are currently the mainstay of targeted immunotherapy for leukaemia. However, their use may be associated with side effects. Infusion-related reactions are quite common and require premedication, which in turn may cause discomfort to patients. Significant interpatient variability in antigen expression and disease subtypes affects treatment outcomes, and the high cost of drugs and their limited availability in some regions are significant barriers to access. There is also a lack of long-term data on their efficacy and safety. These side effects can also pose a risk to the patient. Chemical specificity may cause degradation if stored or handled incorrectly. The active participation of a pharmacist in an interdisciplinary team could increase the effectiveness of treatment and patient safety. Pharmacists, along with doctors and nurses, are key members of the team responsible for patients’ recovery. Their main role is in monitoring therapy, preventing drug interactions, and optimising treatment regimens. However, in our opinion, research should be conducted to clearly understand the role of pharmacists in the use of mAbs to improve leukaemia treatment outcomes. It is this comprehensive approach to therapy that will ensure the patient’s recovery (Figure 15).

## 5. Limitations and Future Directions

This narrative review synthesises the available literature on the use of mAbs in the treatment of various forms of leukaemia and highlights the opportunities for pharmacists in interdisciplinary patient care. While we suggest several pharmacist roles, there are few publications that directly examine the economic, clinical, or humanistic outcomes when clinical pharmacists are involved in the treatment of patients with leukaemia.

Despite these limitations, this review provides a valuable framework for pharmacists, integrating clinical data and practical approaches to optimise monoclonal antibody therapy. However, a significant portion of existing publications focus on short-term outcomes, such as treatment efficacy or side effect profile, with limited emphasis on long-term treatment adherence, drug interactions, and clinical outcomes in patients.

Future studies should focus on evaluating the long-term efficacy and safety of mAbs in different patient groups; studying the impact of pharmacist participation in multidisciplinary teams on clinical outcomes, safety profile, and the economic component of treatment; and developing and implementing innovative models for integrating pharmacists into the care of patients with leukaemia.

## Figures and Tables

**Table 1:** Summary of the properties of mAbs-based medicines and pharmaceutical support of therapy.

Medicinal Product	Drug Group	Risks Associated with Use	Pharmaceutical Care	Premedication
Blincyto (Blinatumomab)	BsAb	Neurotoxicity	Dose adjustment based on patient’s history	Premedication with prednisone: Adults: 100 mg 1 h before the first dose of each cycle. Children: 5 mg only before the first dose in the first cycle and after a break of more than 3 h in the first cycle. Premedication with *dexamethasone*: Adults: 20 mg 1 h before the first dose of each cycle, dose increase and breaks longer than 3 h. Children: 5 mg only before the first dose in the first cycle, before dose increase and after breaks longer than 3 h in the first cycle.
		CRS	Choice of symptomatic therapy	
Besponsa (InO)	Conjugated mAb	Hepatotoxicity	Reduce the number of doses, warn to avoid the use of alkylators	Before administration of the drug, premedication with corticosteroids, antipyretics, and antihistamines is recommended. For patients with circulating lymphoblasts, cytoreduction with a combination of hydroxyurea, steroids, and/or vincristine is recommended before the first administration of the drug, until the peripheral blast level reaches no more than 10,000/mm^3^.
		Infusion-related reactions	Control of premedication, symptomatic treatment	
		TLS	Selection of prophylactic agents	
		Cardiotoxicity	Suspension of the use of drugs that prolong QT	
Mylotarg (GO)	Conjugated mAb	VOD	Fractionated dosing, control of drug interaction with azoles	One hour prior to administration, adult patients should be premedicated with 650 mg of acetaminophen orally and 50 mg of diphenhydramine orally or intravenously, and 30 min before infusion—methylprednisolone 1 mg/kg or an equivalent dose of another corticosteroid. Children should be premedicated with acetaminophen 15 mg/kg (maximum 650 mg), diphenhydramine 1 mg/kg (maximum 50 mg), and methylprednisolone 1 mg/kg orally or intravenously; Additional doses of acetaminophen and diphenhydramine may be administered every 4 h after the initial dose of the previous treatment.
		Infusion-related reactions	Premedication and symptomatic treatment	
		TLS	Preventive measures	
Rituxan (Rituximab)	Chimeric mAb	Infusion-related reactions, cardiotoxicity	Premedication and preventive measures, control of interaction with drugs affecting the cardiovascular system	Premedication with acetaminophen and an antihistamine should be administered prior to each infusion of the drug. For adult patients receiving RITUXAN at an infusion rate of 90 min, the glucocorticoid component of the chemotherapy regimen should be administered prior to infusion. For paediatric patients with mature B-cell NHL/B-ALL, premedication with acetaminophen and H1-antihistamine (diphenhydramine or equivalent) should be administered 30–60 min before the start of each intravenous infusion of the drug. In patients with CLL during treatment and for 12 months after treatment, if necessary.
Arzerra (Ofatumumab)	Human mAb			Patients should receive the following premedication 30 min to 2 h prior to each infusion. Previously untreated CLL: Oral acetaminophen 1000 mg (or equivalent). Oral or intravenous antihistamine (diphenhydramine 50 mg or cetirizine 10 mg or equivalent). Intravenous corticosteroid (prednisolone 50 mg or equivalent).
Gazyva (Obinutuzumab)	Humanised mAb	Infusion reactions associated with the first dose	Premedication	Cycle 1 CLL (days 1 and 2): For all patients: Intravenous glucocorticoid: 20 mg dexamethasone or 80 mg methylprednisolone to be completed at least 1 h prior to infusion. Paracetamol (acetaminophen): 650–1000 mg administered at least 30 min prior to infusion Antihistamine (e.g., 50 mg diphenhydramine) administered at least 30 min prior to infusion. All subsequent cycles For all patients: Acetaminophen 650–1000 mg 30 min before infusion.
		Thrombocytopenia	Control of discontinuation of medications that may provoke the risk of bleeding	
		Hypertensive crisis	Discontinuation of antihypertensive agents	
Campath (Alemtuzumab)	Humanised mAb	Infusion toxicity	Enhanced dosing regimen, premedication	Premedication with diphenhydramine (50 mg) and acetaminophen (500–1000 mg) should be administered 30 min prior to the first infusion and each dose escalation. Administer trimethoprim / sulfamethoxazole twice daily three times a week (or equivalent) as prophylaxis for Pneumocystis pneumonia. Administer famciclovir 250 mg BID or equivalent as prophylaxis for herpes.

**Table 2:** Pharmaceutical profile of mAbs drugs for the treatment of various types of leukaemia.

Name of the Drug	Dosage Form	Container	Excipients	Shelf Life	Storage			
					Refrigerator	Freezing	Light-Protection	Lit. Data
Blincyto (Blinatumomab)	Powder for concentrate and solution for infusion, 38.5 micrograms	Type I glass vial with elastomeric rubber stopper and aluminium seal with flip off cap	Citric acid monohydrate (E330); Trehalose dihydrate; Lysine hydrochloride; Polysorbate 80 (E433); Sodium hydroxide (for pH-adjustment)	Unopened vial, 5 years	2–8 °C	prohibited	Store in the original carton	
	10 mL solution (stabiliser)	Type I glass vial with elastomeric rubber stopper and aluminium seal with flip off cap	Citric acid monohydrate (E330); Lysine hydrochloride; Polysorbate 80 (E433); Sodium hydroxide (for pH adjustment); Water for injections					
Besponsa (InO)	Liophil powder, 1 g	Type I amber glass vial with chlorobutyl rubber stopper and crimp seal with flip off cap	Sucrose Polysorbate 80; Sodium chloride; Tromethamine	Unopened vial, 5 years	2–8 °C	prohibited	Store in the original carton	1 year at ≤25 °C [174]
Mylotarg (GO)	4.5 mg as a lyophilized cake or powder in a single-dose vial for reconstitution and dilution	Amber Type 1 glass vial, with butyl rubber stopper and crimp seal with flip-off cap containing 5 mg GO	Dextran 40; Sucrose; Sodium chloride; Sodium dihydrogen phosphate monohydrate; Disodium hydrogen phosphate anhydrous	Unopened vial, 5 years	2–8 °C	prohibited	Store in the original carton	
Mabthera (Rituxan Rituximab)	Mabthera 500 mg concentrate in solution for infusion	Clear Type I glass vials with butyl rubber stopper	Sodium citrate (E331); Polysorbate 80 (E433); Sodium chloride; Sodium hydroxide (for pH adjustment) (E524); Hydrochloric acid (for pH adjustment) (E507); Water for injections	Unopened vial, 3 years	2–8 °C	prohibited	Store in the original carton	24 h at ≤25 °C [174]
Arzerra (Ofatumumab)	Arzerra 100 mg concentrate in solution for infusion. Arzerra 1000 mg concentrate in solution for infusion	Clear Type I glass vial with a bromobutyl rubber stopper and aluminium over-seal, containing 5 mL of concentrate in solution for infusion	Arginine; Sodium acetate (E262); Sodium chloride; Polysorbate 80 (E433); Edetate disodium (E386); Hydrochloric acid (E507) (for pH-adjustment); Water for injections	Unopened vial, 3 years	2–8 °C	prohibited	Store in the original carton	
Gazyvaro (Obiuntuzumab)	1000 mg concentrate in solution for infusion [174]	Amber Type 1 glass vial, with butyl rubber stopper	Histidine Histidine hydrochloride monohydrate Trehalose dihydrate Poloxamer 188 Water for injections	Unopened vial, 3 years	2–8 °C	prohibited	Store in the original carton	24 h at ≤25 °C [174]
Campath (Alemtuzumab)	Single-use transparent glass vials containing 30 mg of alemtuzumab in 1 mL of solution	A sterile, clear, colourless, isotonic solution (pH 6.8–7.4) in a single-dose vial for intravenous administration	30 mg of alemtuzumab, 8.0 mg of sodium chloride, 1.44 mg of disodium phosphate, 0.2 mg of potassium chloride, 0.2 mg of monobasic potassium phosphate, 0.1 mg of polysorbate 80, and 0.0187 mg of disodium edetate dihydrate	Unopened vial, 3 years	2–8 °C	prohibited	Store in the original carton	1 month at 30 ± 2 °C and 3 months at 25 ± 2 °C [174]

**Table 3:** Stability of prepared infusion solution [174,175,176,177,178].

Name of the Drug	Container Type	Concentration	Temperature Regime	Storage Time
Blincyto (Blinatumomab)	vials	12.5 μg/ml	2–8 °C	24 h
			23–27 °C	4 h
	polyolefin and ethylene vinyl acetate infusion bags	0.26 μg/mL	2–8 °C	10 days
			23–27 °C	96 h
Besponsa (InO)	Use immediately after preparation.			
Mylotarg (GO)	Use immediately after preparation.			
Mabthera (Rituxan Rituximab)	glass vials	10 mg/mL	23–32 °C	21 days
	partially used vials		2–4 °C	28 days
			25 °C	15 days
	dilution of the solution in 0.9% NaCl in polyethylene bags.	1 mg/mL	2–8 °C	31 days
			23–27 °C	30 days
			28–32 °C	14 days
	polypropylene syringes		2–8 °C	31 days
	polyolefin bags		2–4 °C	28 days
			25 °C	15 days
	polypropylene syringes	120 mg/mL	2–8 °C	28 days
			30 °C	24 h
		1–4 mg/mL in 0.9% NaCl	2–8 °C	30 days
			<30 °C	24 h
		1–4 mg/mL in 5% glucose	2–8 °C	24 h
			25 °C	12 h
For biosimilars	polyolefin bags	1 mg/mL	2–8 °C	up to 180 days
Arzerra (Ofatumumab)	in solutions prepared on the basis of a 0.9% sodium chloride solution	0.3 mg/mL and 2 mg/mL	25 °C	48 h
Gazyvaro (Obiuntuzumab)	PVC and polyolefin bags	0.4–20 mg/mL	2–8 °C	24 h
			<30 °C	48 h
Campath (Alemtuzumab)	in solutions prepared from 0.9% sodium chloride solution or 5% glucose solution	0.1 mg/mL	2–8 °C	8 h

## Data Availability

No new data were created or analysed in this study. Data sharing is not applicable to this article.

## Abbreviations

The following abbreviations are used in this manuscript:

ALL	Acute lymphocytic leukaemia
AML	Acute myeloid leukaemia
B-ALL	B-lineage acute lymphocytic leukaemia
B-CLL	B-cell chronic lymphocytic leukaemia
BsAb	Bispecific antibody
CLL	Chronic lymphocytic leukaemia
CML	Chronic myeloid leukaemia
CRS	Cytokine release syndrome
FDA	U.S. Food and Drug Administration
GO	Gemtuzumab ozogamicin
ICER	Institute for Clinical and Economic Review
ILD	Interstitial lung disease
InO	Inotuzumab ozogamicin
NICE	National Institute for Health and Care Excellence
mAbs	Monoclonal antibodies
MRD	Minimal residual disease
Ph	Philadelphia
PML	Progressive multifocal leukaemia
R/R	Relapsed or refractory
TLS	Tumour lysis syndrome
TT	Targeted therapy
VOD	Veno-occlusive liver disease
WHO	World Health Organisation
